# Current Strategies to Improve the Bioactivity of PEEK

**DOI:** 10.3390/ijms15045426

**Published:** 2014-03-28

**Authors:** Rui Ma, Tingting Tang

**Affiliations:** Shanghai Key Laboratory of Orthopedic Implants, Department of Orthopedic Surgery, Shanghai Ninth People’s Hospital, Shanghai Jiao Tong University School of Medicine, Shanghai 200011, China; E-Mail: shandongmarui@126.com

**Keywords:** polyetheretherketone, bioactivity, surface modification, coating, composite

## Abstract

The synthetic thermoplastic polymer polyetheretherketone (PEEK) is becoming a popular component of clinical orthopedic and spinal applications, but its practical use suffers from several limitations. Although PEEK is biocompatible, chemically stable, radiolucent and has an elastic modulus similar to that of normal human bone, it is biologically inert, preventing good integration with adjacent bone tissues upon implantation. Recent efforts have focused on increasing the bioactivity of PEEK to improve the bone-implant interface. Two main strategies have been used to overcome the inert character of PEEK. One approach is surface modification to activate PEEK through surface treatment alone or in combination with a surface coating. Another strategy is to prepare bioactive PEEK composites by impregnating bioactive materials into PEEK substrate. Researchers believe that modified bioactive PEEK will have a wide range of orthopedic applications.

## 1. Introduction

Aging related aggravation and increases in accidental injuries have resulted in a sharp increase in the incidence of many diseases related to the bone and joint system, including fracture, vertebral degeneration, arthritis, bone tumors and tuberculosis. Orthopedic surgery using implants is now the main method to restore the structure and function of damaged bones and joints. Orthopedic implant materials commonly used in the clinic mainly include metals, ceramics, polymers and composites.

Metallic implants (e.g., gold, tantalum (Ta), stainless steel, shape memory alloy (NiTi), titanium (Ti) alloy, cobalt chromium (Co-Cr) alloy), have been widely used in the clinic either as permanent prostheses (such as the hip prosthesis, dental implants, *etc*.), or as temporary implants (such as plates, pins, screws and rods for the fixation of bone fractures). Metals can provide favorable mechanical strength, excellent friction-resistance and non-toxic properties [1–3]; however, some notable disadvantages have hindered their more widely medical applications [4–8]. Their high strength and elastic modulus that do not match those of normal human bone tissues can cause a stress shielding effect on the peri-implant bones, which will led to adsorption of adjacent bone tissues and cause prosthetic loosening. The radiopacity of metals causes artifacts in computed tomography (CT) images and limits the ability to examine the patient with magnetic resonance imaging (MRI). The long-term presence of metals *in vivo* can trigger allergic tissue reactions and initiate osteolysis.

Ceramics, including metallic oxides (e.g., Al_2_O_3_, MgO), calcium phosphate (e.g., hydroxyapatite (HA), tricalcium phosphate (TCP), octacalcium phosphate (OCP)) and glass ceramics (e.g., bioglass, ceravital), have received a great deal of attention from material scientists. Among these ceramics, metallic oxides are inert ceramics, and calcium phosphate and glass ceramics are bioactive ceramics that are commonly used at present. Bioactive ceramics exhibit favourable non-toxicity and corrosion-resistance, good biocompatibility and bioactivity. However, the mechanical properties of these materials, including their low fracture toughness and ductility, high elastic modulus and brittleness, cannot meet the demands of the load-bearing applications [9].

Polymers, such as ultra-high molecular weight polyethylene (UHMWPE), polytetrafluoroethylene (PTFE), polymethyl methacrylate (PMMA), polylactide (PLA), polyglycolide (PGA) and polyhydroxybutyrate (PHB), are widely used in various biomedical applications. However, only a limited number of polymers have been used for bone replacement purposes because they tend to be too flexible and too weak to meet the mechanical demands as orthopedic implants [10–12]. Besides, they may absorb liquids and swell, leach undesirable products and may be affected by sterilization process [10].

Polyetheretherketone (PEEK) is a semi-crystalline linear polycyclic aromatic thermoplastic that was first developed by a group of English scientists in 1978 [13]. In the 1980s, PEEK was commercialized for industrial applications, such as aircraft and turbine blades [14]. By the late 1990s, PEEK became an important high-performance thermoplastic candidate for replacing metal implant components, especially in orthopedic and traumatic applications [15]. PEEK was commonly used in vertebral surgery as a material of the interbody fusion cage [16–18]. With the emergence of carbon fiber reinforced PEEK (CF/PEEK), this new composite material was exploited for fracture fixation and femoral prosthesis in artificial hip joints [19,20]. Over the past few years, PEEK and its composites have attracted a great deal of interest from material scientists and orthopedists.

PEEK, a member of the polyaryletherketone family, has an aromatic molecular backbone, with combinations of ketone and ether functional groups between the aryl rings [20]. This special chemical structure makes PEEK exhibit stable chemical and physical properties [13,15,20–26]: it is wear-resistant and stable at high temperatures [13]; it is resistant to attack by all substances apart from concentrated sulfuric acid [15,20]; it remains stable in sterilization processes [21]. Besides, PEEK exhibits good biocompatibility *in vitro* and *in vivo*, causing neither toxic or mutagenic effects nor clinically significant inflammation [22–25]. More importantly, the mechanical properties of PEEK are close to that of human cortical bone [26]. For example, the elastic modulus of PEEK is approximately 8.3 GPa, which is close to that of human cortical bone (17.7 GPa) and much lower than that of Ti alloy (116 GPa) and Co–Cr alloy (210 GPa) [12]. However, PEEK is biologically inert [26,27], which has limited its potential applications. Therefore, improving the bioactivity of PEEK is a significant challenge that must be solved to fully realize the potential benefits. Currently, two major strategies have been used to improve the bioactivity of PEEK, including surface modification and composite preparation, which will be reviewed in our present article.

## 2. Surface Modification

**Figure 1.**
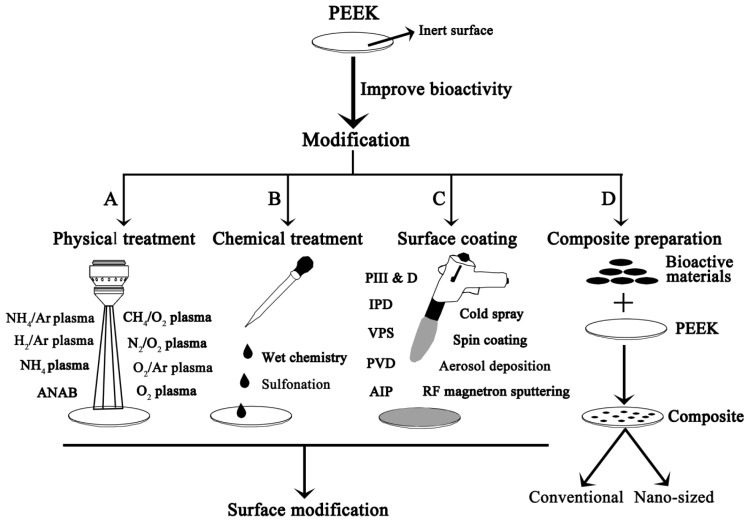
Scheme of current strategies to improve the bioactivity of PEEK.

Although PEEK is always physically and chemically stable, it can be modified by some kind of physical or chemical treatments. The commonly-used physical treatments are plasma modifications (such as oxygen (O_2_) plasma, ammonia (NH_4_) plasma, nitrogen and oxygen (N_2_/O_2_) plasma, methane and oxygen (CH_4_/O_2_) plasma, oxygen and argon (O_2_/Ar) plasma, ammonia/argon (NH_4_/Ar) plasma, and hydrogen/argon (H_2_/Ar) plasma), and accelerated neutral atom beam (ANAB) (Figure 1A). The chemical treatments were rare. Only wet chemistry modification or sulfonation treatment can chemically modify the surface of PEEK (Figure 1B). Besides, some materials can be coated onto PEEK to impose bioactive effects using various methods, including cold spray technique, radio-frequency (RF) magnetron sputtering, spin coating techniques, aerosol deposition (AD), ionic plasma deposition (IPD), plasma immersion ion implantation and deposition (PIII&D), electron beam deposition, vacuum plasma spraying (VPS), physical vapor deposition (PVD), and arc ion plating (AIP) (Figure 1C). Surface treatment alone or in combination with surface coating can greatly improve the bioactivity of PEEK.

### 2.1. Surface Treatment

#### 2.1.1. Physical Treatment

Plasmas are ionized gases that can be produced in a closed reactor system containing a low pressure gas mixture by excitation with electro-magnetic waves [27]. The reactive particles generated in this way can interact with the surface of the biomaterial placed in the reactor and modify its physical and chemical surface properties without changing the mechanical, electrical and optical properties of the material that are relevant to its application [27,28]. The method of plasma modification has been used to modify PEEK material for a long time. Briem *et al.* [27] treated PEEK surface with two plasma process (a microwave plasma in NH_4_/Ar and a downstream microwave plasma in H_2_/Ar), and investigated the proliferation and differentiation of primary fibroblasts and osteoblasts on plasma-treated PEEK. They found that the osteogenic activity of cells on treated PEEK was comparable to that of tissue culture polystyrene (TCPS), and a reproducible stimulation and suppression of cell proliferation could be achieved by the methods of plasma modification. Ha *et al.* [29] treated PEEK with N_2_/O_2_ low-pressure plasma to improve the bioactivity of PEEK. Cell testing with osteoblastic cell lines (MC3T3-E1) showed that plasma-treated PEEK had no disadvantageous effects on cell viability. After 24 days of immersion in a calcium and phosphate-saturated solution, a carbonate-containing calcium phosphate layer with a thickness of up to 50 μm was formed on the surface of plasma-treated PEEK. Compared with the untreated PEEK, the cell viability on the plasma-treated PEEK coated with calcium phosphate was significantly increased. Awaja *et al.* [30,31] treated PEEK with RF plasma with a mixture of CH_4_/O_2_ gases to modify the surface of PEEK. They found that the treatment with CH_4_/O_2_ gases resulted in a significantly higher bond strength than untreated samples [30]. Using a plasma immersion ion implantation and deposition (PIII&D) technique with a CH_4_/O_2_ gas mixture, they detected the deposition of oxygen-rich nanofilms on PEEK with a high surface energy, which greatly improved cell adhesion [31]. They also found there are strong correlations between cell adhesion and the water contact angle, the polar component of surface energy, and to a lesser extent oxygen concentration of the PEEK surfaces [31]. Brydone *et al.* [32] fabricated novel nanopatterned PEEK rods, etched these PEEK rods with O_2_ plasma to improve their bioactivity, and then implanted them into a femoral defect rabbit model. Animal testing results proved that this nanopatterned PEEK etched by oxygen plasma exhibited potential osteoinductivity *in vivo*. Waser-Althaus [33] applied the O_2_/Ar or NH_4_ plasma to treat the PEEK surface. They demonstrated an increased adhesion, proliferation, and osteogenic differentiation of adipose tissue-derived mesenchymal stem cells (adMSC) on plasma-treated PEEK, and a doubled mineralization degree on 50 W plasma-treated PEEK relative to the 10 W was observed, indicating the osteogenic differentiation was dependent on the plasma power.

A novel ANAB technique employing intense directed beams of neutral gas atoms (comprised of van der Waals bonded argon atoms) with average energies that could be controlled resulted in a controllable nanometer scale texturing of the surface to a depth of no more than 5 nm [34,35]. Khoury *et al.* [34] employed the ANAB technique to enhance the surface bioactivity of PEEK without modification of surface chemistry and without the addition of bioactive substances. *In vitro* experiments demonstrated that the ANAB-treated PEEK fostered enhanced growth of human fetal osteoblast cells (hFOB) compared with untreated PEEK as evidenced by cell proliferation assays and microscopy. Using a rat calvarial defect model, they revealed that ANAB-treated PEEK enhanced osteointegration, with bone tissue formation only evident on the ANAB-treated PEEK.

#### 2.1.2. Chemical Treatment

Wet surface chemistry has been used to chemically modify PEEK to create a series of surface-functionalized PEEKs. They are hydroxylated polymer (PEEK–OH) obtained by reduction, carboxylated polymer (PEEK–NCO) prepared by coupling a diisocyanate reagent to PEEK–OH, aminated polymer (PEEK–NH_2_) acquired by hydrolysis of PEEK–NCO, and aminocarboxylated polymers (PEEK–GABA and PEEK–Lysine) resulting from the coupling of aminoacids to PEEK–NCO [36,37]. These chemical modifications promoted higher levels of fibronectin covalently fixed and/or adsorbed on various treated PEEK compared with untreated PEEK. The carboxylated polymer and aminated polymer promoted the adhesion and growth of CaCo_2_ cells (cell line derived from a human colon adenocarcinoma) in the presence of serum.

By sulfonation and subsequent water immersion, a 3D porous and nano-structured network with bio-functional groups is produced on PEEK to prepare two kinds of sulfonation-treated PEEK (SPEEK) samples (SPEEK-W (water immersion and rinsing after sulfonation) and SPEEK-WA (SPEEK-W with further acetone rinsing)), and the *in vitro* cellular behavior, *in vivo* osseointegration, and apatite-forming ability of the sulfonation-treated PEEK were systematically investigated [38]. The results showed that SPEEK-WA induced pre-osteoblast functions including initial cell adhesion, proliferation, and osteogenic differentiation *in vitro* as well as substantially enhanced osseointegration and bone-implant bonding strength *in vivo* and apatite-forming ability. Although SPEEK-W has a similar surface morphology and chemical composition as SPEEK-WA, its cytocompatibility is inferior due to residual sulfuric acid.

### 2.2. Surface Coating

Various materials have been deposited on the surface of PEEK, including hydroxyapatite (HA), titanium (Ti), gold, titanium dioxide (TiO_2_), diamond-like carbon (DLC), and *tert*-butoxides. The bioactivity of PEEK can be greatly enhanced by these surface coatings.

The most commonly-used bioactive material as coating of PEEK is HA. HA (chemical formula Ca_10_(PO_4_)_6_(OH)_2_) is the most widely used calcium phosphate-based bioceramic, which is the closest pure synthetic equivalent to human bone mineral [39]. Numerous studies have consistently shown that HA typically exhibits excellent biocompatibility, bioactivity, and osteoconduction *in vivo* [40–42]. Lee *et al.* [43] used a cold spray technique to fabricate HA-coated PEEK and evaluated its bioactivity *in vitro* and *in vivo. In vitro* tests indicated that the adhesion, viability and osteoblast differentiation of human bone marrow mesenchymal stem cells (hBMSCs) were improved on HA-coated PEEK compared with the uncoated one. For *in vivo* tests, these authors implanted HA-coated PEEK cylinders into a rabbit ilium model with uncoated PEEK as control and demonstrated that HA-coated PEEK promoted implant osteointegration with the surrounding bone using micro-computed tomography (micro-CT) and histomorphometric analysis. Barkarmo *et al.* [44] fabricated nanocrystalline HA-coated PEEK with a spin coating technique and inserted the cylinder implants into the femurs of rabbits with uncoated cylinders as controls. The nano-HA coated PEEK cylinders exhibited a higher mean bone-implant contact than uncoated cylinders, indicating that nano-HA coated PEEK promoted osteointegration. Highly dense and well-adhered HA coating could be developed on PEEK using aerosol deposition (AD) without thermal degradation of PEEK [45]. *In vitro* and *in vivo* bioactivity of PEEK, in terms of cell adhesion, morphology, proliferation, differentiation, and bone-to-implant contact ratio, were remarkably enhanced by the HA coating. In another study [46], HA coatings were deposited onto PEEK surfaces using RF magnetron sputtering. Before HA deposition, a yttria-stabilized zirconia (YSZ) coating layer was deposited onto PEEK substrates to prevent degradation of PEEK substrates and the coating-substrate interface. Then, the HA/YSZ coated PEEK was heat treated using microwave and hydrothermal annealing to form the crystalline HA. Cell tests showed a significant increase in initial cell attachment and growth on the microwave-annealed HA/YSZ-coated PEEK compared with uncoated PEEK and amorphous HA. Jung *et al.* [47] prepared a PEEK/Mg composite with a Mg content of 30 vol % by compression molding process, then they treated the composite in a specifically prepared aqueous solution for HA coating, which led to the formation of an HA coating layer only on Mg particles exposed to the surfaces of the composite. The HA-coated PEEK/Mg composite was proved to exhibit enhanced *in vitro* bio-corrosion resistance and bioactivity (with more attached MC3T3-E1 cells exhibiting active cytoskeletal extension and more significant proliferation) compared with pure PEEK and the uncoated PEEK/Mg composite.

Titanium (Ti) is the most widely used implant material for load-bearing dental and orthopedic applications because of its excellent mechanical and biological properties [48]. Therefore, Ti is an appropriate candidate as the coating material for PEEK. Chang Yao *et al.* [49] studied osteoblast adhesion on PEEK coated with either Ti or gold using the ionic plasma deposition (IPD) process, which created a nanostructured surface (with features below 100 nm). Compared with the commonly used Ti and uncoated PEEK, PEEK coated with either Ti or gold significantly increased osteoblast adhesion and spreading. They attributed the increased cell adhesion to the nanometer surface roughness and the changed surface wettability. Cook *et al.* [50] applied plasma vapor deposition (PVD) to coat Ti onto PEEK surface and placed coated PEEK and uncoated PEEK cylindrical implants into the femurs of mongrel dogs. The histological evaluation and mechanical evaluation revealed that the Ti-coated specimens had significantly higher percentages of bone contact than the uncoated specimens at both 4 and 8 weeks, and the uncoated implants had significantly higher shear strength values than the coated implants at 4 weeks. Han *et al.* [51] coated Ti onto PEEK using electron beam (e-beam) deposition process, which produced a dense, uniform film on the substrate at a low temperature. The *in vitro* cellular responses of the samples were assessed in terms of cell attachment, proliferation, and osteoblastic differentiation, and the *in vivo* bone conductivity was examined by measuring the bone-to-implant contact (BIC) ratio using a rabbit tibial defect model. The level of proliferation and differentiation of the MC3T3-E1 cells was more than doubled after Ti was coated onto the PEEK surface, and the *in vivo* animal tests showed that the Ti-coating PEEK implants had a much higher BIC ratio than the pure PEEK implants. In one study [52], CF/PEEK was coated with Ti by vacuum plasma spraying (VPS) process and chemically treated in sodium hydroxide (NaOH) solution. A carbonate-containing calcium phosphate layer was formed on the NaOH-treated Ti-coated CF/PEEK surface during immersion in simulated body fluid (SBF), whereas no calcium phosphate precipitation occurred on the untreated PEEK surfaces. In another study [53], CF/PEEK screws were coated with Ti using two different techniques, VPS and PVD. The coated CF/PEEK was implanted into the tibia of sheep with uncoated CF/PEEK as the control. The results showed that Ti-coated CF/PEEK screws significantly improved bone deposition and removal torque compared with uncoated screws, whereas no statistical difference was detected between VPS and PVD coating types.

Titanium dioxide (TiO_2_) material has been demonstrated with good biocompatibility, bioactivity, hydrophilicity, and corrosion resistance [54,55]. Anatase phase (A-TiO_2_) and/or rutile phase (R-TiO_2_) can be deposited onto PEEK substrate by an arc ion plating (AIP) technique following three steps (argon ion bombardment, bottom titanium layer deposition and TiO_2_ coating deposition) at a low deposition temperature without damaging PEEK substrate, while providing satisfactory film adhesion [56–58]. From results of cell adhesion, proliferation and osteo-differentiation abilities, the authors concluded that the TiO_2_-coated PEEK exhibited better osteoblast compatibility than bare PEEK and R-TiO_2_/PEEK exhibited better osteoblast compatibility than A-TiO_2_/PEEK [56,57]. Surface roughness and hydrophilicity of the AIP-TiO_2_ films were found to be responsible for significant osteoblast cell growth and the presence of negatively charged hydroxyl groups on R-TiO_2_ contributed to its better cytocompatibility than A-TiO_2_. In SBF immersion test, TiO_2_-coated PEEK presented enhanced HA growth with the crystallinity and film thickness of the grown HA layer proportional to immersion time, and R-TiO_2_/PEEK exhibited superior ability to induce HA formation due to the pre-absorbed negatively charged groups on R-TiO_2_ coating surface [58]. Han *et al.* [59] created a uniform nanoporous TiO_2_ layer with a pore diameter of ~70 nm by anodizing a Ti film, then deposited the created TiO_2_ onto a PEEK substrate via e-beam evaporation technique, and immersed the specimens in a bone morphogenetic protein-2 (BMP-2) solution to immobilize BMP-2. The *in vitro* cell tests and *in vivo* animal tests demonstrated that the nanoporous TiO_2_ surface immobilized with BMP-2 could significantly enhance the cell attachment, proliferation, differentiation of MC3T3-E1 cells, and the osseoconductivity of PEEK implants. The BMP-immobilized PEEK coated with nanoporous TiO_2_ showed much higher BIC ratio (60%) than the bare PEEK (30%), PEEK coated with nanoporous TiO_2_ (50%) and even BMP-immobilized PEEK without coating (32%).

Except for the commonly-used coating materials (HA, Ti and TiO_2_), some other infrequent materials were used as coating materials on PEEK. Chu *et al.* [26] successfully coated PEEK with diamond-like carbon (DLC) by plasma immersion ion implantation and deposition technique. A cell viability assay, scanning electron microscopy (SEM) and real-time polymerase chain reaction (PCR) analysis indicated that osteoblast attachment, proliferation and differentiation were better on DLC-coated PEEK than on bare PEEK. In another study [60], vapor of zirconium or titanium tetra (*tert*-butoxides) was deposited on the surface of PEEK at room temperature in a process reminiscent of deposition and partial thermolysis of metal alkoxides on oxide surfaces. Controlled thermolysis of the deposited alkoxide gives the metal a mixed oxide-alkoxide layer, which reacts with solutions of phosphonic acids to attach monolayer films of phosphonates, several of which are shown to significantly enhance osteoblast attachment and spreading compared with the untreated surface.

In addition to coating various materials onto PEEK, PEEK material can also be coated onto other materials. Using the electrophoretic deposition (EPD) method, PEEK and PEEK/bioglass particles were coated onto shape memory alloy (nickel and titanium, NiTi) wires [61] or on two phase (α + β) Ti–6Al–7Nb titanium alloy substrates [62] with a uniform coating surface and negligible microcracking or porosity. As corrosion protective layers, the PEEK and PEEK/bioglass coatings were able to impede the leakage of ions in contact with body fluids. In particular, the bioglass containing coatings improved the bonding of bone or soft tissue to the implant. After immersion of PEEK/bioglass coated NiTi in SBF, hydroxyapatite layers formed on the surface of the coated specimens after one week.

## 3. PEEK Composites

Some ceramics, such as HA, TCP, calcium silicate (CS), bioglass, glass-ceramic A-W, are referred to as bioactive materials due to their ability to spontaneously bond to living bone, and these materials are already used as bone substitutes with important clinical applications [9]. Unfortunately, these bioactive materials exhibit a lower fracture toughness and higher elastic modulus compared with human cortical bone [9,40]. Although PEEK can provide favourable mechanical properties, its native inertness prevents good bonding with surrounding bone tissues. Thus, impregnating bioactive materials into PEEK has become one attractive strategy to improve the bioactivity of PEEK while maintaining its mechanical properties. The PEEK composites were classified into two kinds by the size of the impregnating bioactive materials: the conventional PEEK composites and the nano-sized (<100 nm) PEEK composites (Figure 1D). The reported PEEK composites that are related to the bioactivity of the composites are shown in Table 1.

### 3.1. Conventional PEEK Composites

With good biocompatibility, bioactivity and osteoconduction, HA is not only used as common coating material for PEEK, but also as common filler material to prepare PEEK composite. Several studies has investigated the mechanical properies of the HA incorporated PEEK composite (HA/PEEK). Khor’s research group [63,64] fabricated a HA/PEEK composite with an HA content of up to 40 vol % via a process of melt compounding, granulation and injection-molding. Increasing the HA content resulted in increasing of the tensile modulus and microhardness, but decreasing the tensile strength and strain to fracture. These authors also found that PEEK with 30 vol % HA exhibited an elastic modulus within the range of human cortical bone. All of the specimens (5, 10, 20, 30, and 40 vol % HA) survived cyclic loading at 50% ultimate tensile strength and exhibited a high estimated fatigue strength at 1 million cycles. Similar results can be found in the report by Converse and co-workers [65]. However, Khor and co-workers [63,64,66,67] found that the spray-dried spherical HA particles in conventional or micro-sized HA/PEEK (μm-HA/PEEK) composites could debond from the PEEK matrix during long-term loading due to the poor interfacial adhesion. Fatigue damage of μm-HA/PEEK composites began with filler matrix interface failure, followed by initiation and propagation of matrix cracks from the filler-matrix debonding site, and subsequent development of longer matrix cracks from shorter cracks caused final failure [64]. The biocompatibility and bioactivity of HA/PEEK composites has been studied by several researchers. Zhang *et al.* [68] manufactured HA/PEEK composites via the selective laser sintering (SLS) technique and evaluated cell attachment, morphology, proliferation and differentiation using primary human osteoblast cells. They found that the SLS-treated HA/PEEK supported osteoblast growth and that composites with higher HA contents exhibited enhanced cell proliferation and osteogenic differentiation (increased ALP activity, and produced more osteocalcin), compared to thermanox (TMX) and polyvinyl chloride (PVC). Ma *et al.* [69,70] successfully prepared an HA/PEEK composite via an *in situ* synthetic process. The composite exhibited an excellent improvement in mechanical properties and bonding between HA and PEEK. Subsequently, to examine the possible adverse effects of the residual organic chemicals from the *in situ* synthesis process, the biocompatibility of the material was investigated. The *in situ* synthesized composite exhibited good biocompatibility without toxicity, and the composite with 5.6 vol % HA exhibited satisfactory bioactivity without compromising its excellent mechanical performance. Bioactive materials can form a bone-like apatite layer on their surfaces *in vivo* and bond to bone through this apatite layer [9]. Thus, the bone-bonding ability of a material is often evaluated by examining the ability of apatite to form on its surface in a simulated body fluid (SBF) with ion concentrations nearly equal to those of human blood plasma [71]. Yu *et al.* [72] prepared HA/PEEK composite by mixing, compaction, and pressureless sintering process, and evaluated the bioactivities of HA/PEEK composites with 10, 20, 30 and 40 vol % HA by immersing the composite disks in SBF for 4 weeks. Pure PEEK exhibited no significant changes on its surface after 28 days of immersion, and the surface of composite with 40 vol % HA was covered by a layer of bone-like apatite just after 3 days of immersion, while 10 vol % HA was covered after 28 days. The growth rate increased with HA volume fraction, suggesting that the bioactivity of the HA/PEEK composite increased with increasing HA content in the composite. Invibio has released an HA-filled PEEK compound with microscale HA particles called “PEEK-OPTIMA HA enhanced polymer” for use in implants [73]. PEEK-OPTIMA HA enhanced biomaterial provides excellent mechanical properties and performance, proven biocompatibility, a modulus similar to cortical bone, reducing stress shielding and a high degree of radiolucency that allows for clear fusion assessment. Within four weeks of implantation Invibio “PEEK-OPTIMA HA enhanced polymer” demonstrated enhanced bone apposition compared to PEEK-OPTIMA^®^ Natural, in a pre-clinical *in vivo* study using a sheep model. Within 12 weeks of implantation the bone apposition levels are maintained with the new grade.

To favour bone-in-growth to the composites and make strongly bonded implant/bone interface, some measures were adopted to prepare porous HA/PEEK composite. Abu Bakar *et al.* [66] prepared 20 vol % HA/PEEK with porosity of 60% and pore size ranging from 300 to 600 mm by leaching of particulate technique employing a suitable pore-forming agent, and implanted these materials into the distal metaphyseal femur in pigs to evaluate the biological responses and tissue in-growth of the material. Histological studies revealed the presence of fibro-vascular tissue within the pores at 6 weeks and mature bone formation at 16 weeks after implantation. Using an SLS rapid prototyping system, porous HA/PEEK composite scaffolds starting with 10 wt % HA to 40 wt % HA have been produced by Tan *et al.* [74,75]. Both the microporosity and macroporosity of the scaffolds showed that highly porous HA/PEEK scaffolds could be obtained. The immersion of HA/PEEK scaffolds in SBF demonstrated the bioactivity of the specimens by the precipitation of apatite-layers. Cell culture of fibroblast cell lines on HA/PEEK scaffolds demonstrated positive cell adhesion and growth. However, the attainable level of porosity was limited to 70%–74% which was dependent on both the reinforcement level and laser power. To overcome this disadvantage, Converse *et al.* [76] fabricated HA whisker-reinforced PEEK with high levels of porosity (75%–90%) and HA whisker reinforcement (0–40 vol %) using a powder processing, followed by compression molding and particle leaching, but neither *in vitro* nor *in vivo* tests related to this scaffold have been reported. As the mechanical properties may be compromised with increasing the HA content, determining the appropriate HA content to attain both satisfying mechanical properties and bioactivity is crucial in the fabrication of HA/PEEK composites. More studies should be focused on this point.

Apart from HA, other bioactive materials were also used to make bioactive PEEK composites, including strontium-containing hydroxyapatite (Sr-HA), calcium silicate, glass fibers, bioglass, and β-tricalcium- phosphate (β-TCP). Wong *et al.* [77] developed Sr-HA/PEEK composites with Sr-HA content ranging from 15–30 vol % by a compression molding technique. The addition of Sr-HA outperformed HA in increasing the bioactivity of the composite based on a qualitative comparison of apatite formation in SBF and the quantitative measurement of MG-63 cell-mediated mineralization via alizarin red staining *in vitro*. However, no difference was observed in the cell proliferation and ALP activity between Sr-HA/PEEK and HA/PEEK composites at each time point. Kim *et al.* [78] fabricated CS-reinforced PEEK composite (CS/PEEK) with 0–50 vol % CS and soaked specimens in SBF with pure PEEK as the control. Except for pure PEEK, all of the CS-containing composites promoted apatite formation on their surfaces, exhibiting the potential to bond to living bone. The time required for the induction of apatite formation on the composite surfaces decreased with increasing CS content. The mechanical properties of the samples after soaking in SBF did not significantly decrease compared with samples that were not exposed to SBF. Considering both mechanical properties and bioactivity, these authors selected 20 vol % CS/PEEK as a promising implant material. Glass fiber/PEEK (GPEEK) composites were developed using PEEK and 10% randomly chopped E-glass fibers, and the cell proliferation, ALP activity and osteocalcin production on GPEEK using MG-63 cells were analysed [79,80]. GPEEK supported proliferation, ALP activity and osteocalcin production *in vitro*, suggesting that GPEEK could improve the growth and differentiation of bone cells. β-TCP was also incorporated into PEEK, and β-TCP was not found to improve the bioactivity of PEEK. Wilmowsky’s research group [81] compared human osteoblast proliferation on pure PEEK, PEEK/1 wt % carbon and PEEK/1 wt % carbon/10 wt % β-TCP. The results showed that PEEK composites containing 10 wt % β-TCP did not improve the proliferation of osteoblasts *in vitro*. They also compared cell growth among pure PEEK, PEEK/1 wt % carbon, PEEK/1 wt % carbon/10 wt % β-TCP and PEEK/1 wt % carbon/ 10 wt % bioglass fabricated by laser sintering [82]. Cell proliferation and cell viability tests using hFOB cells showed that none of these composites induced cytotoxicity. The rates of proliferation of human osteoblasts growing on PEEK/1 wt % carbon/10 wt % bioglass were significantly higher than those on the other groups. However, some evidence indicated the inhibitory effect of β-TCP/PEEK on osteoblast proliferation. Petrovic *et al.* [83] studied the effect of PEEK containing 5, 10, 20 and 40 wt % β-TCP processed by injection molding on normal human osteoblast (NHOst) cells. The results showed that the proliferation rates of NHOst cells growing on β-TCP/PEEK were lower than those on pure PEEK, but β-TCP/PEEK showed no concentration-dependent decrease in cell proliferation compared with the pure PEEK. Von Wilmowsky also observed a lower cell viability and proliferation on β-TCP/PEEK compared with pure PEEK [82]. These authors suggested that a shift in the pH of the cell culture medium resulting from the degradation of the β-TCP compound may contribute to the inhibitory effect of β-TCP/PEEK on cell proliferation. Therefore, more detailed investigations are required to understand the effects of β-TCP/PEEK on osteoblasts.

### 3.2. Nano-Sized PEEK Composites

Conventional HA/PEEK composite may not bear long-term critical loading due to debonding between HA filler and PEEK matrix; which has been illustrated in detail in part 3.1. Nanotechnology was applied by material scientists to overcome this problem. Wang *et al.* [84,85] prepared HA/PEEK nanocomposites by a compounding and injection molding process. They found that this novel HA/PEEK nanocomposite exhibited satisfactory mechanical properties and a high surface HA content. More importantly; no debonding occurred between the well-dispersed HA nanoparticles and the PEEK matrix. However; the agglomeration of HA nanoparticles became severe as the HA content increased over 10 vol %. This process may be related to the high viscosity of the PEEK matrix at high temperatures during the manufacturing process [84]. Other studies also found a general tendency for nanoparticles to aggregate during the fabrication of nanoparticle-reinforced thermoplastics [84–87]. It was difficult to uniformly disperse the nanoscale powders in a viscous polymer matrix using the conventional methods [40]. To overcome the agglomeration of HA nanoparticles during manufacturing; these authors adopted an *in situ* synthetic process to prepare HA/PEEK nanocomposites [88]. In this *in situ* synthetic process, HA particles were first mixed into PEEK oligomers with short chains and a low viscosity and good wetting and contact were achieved between HA and PEEK. Then, continuing polymerization increased the molecular weight of the PEEK oligomers on the HA surface and the oligomers were firmly wrapped on the HA surface. The strong bonding between HA and PEEK has been attributed mainly to physical factors such as the mechanical interlock between PEEK molecules and the HA surface.

In native bone tissues, bone cells are exposed to substrates and structures with nanoscale features, such as extracellular matrix (ECM) proteins, minerals and pores in membranes and tissues [89]. By mimicking this nanotopography through the fabrication of nano-sized materials, researchers hope to enhance bone cell growth and tissue integration [90]. When the feature size of a material is decreased from micrometers to nanometers, the material exhibits several unique characteristics, including a very high surface area to volume ratio, flexible surface functionality and superior mechanical performance, including stiffness and tensile strength [91,92]. Webster and co-workers [93–97] have conducted a great deal of research on the bioactivity and biocompatibility of nanomaterials. They found that nanostructured materials may promote osteoblast adhesion, proliferation, differentiation, and stimulate new bone growth compared to conventional materials.

Therefore, developing PEEK composites reinforced with nano-sized bioactive materials is a promising strategy for obtaining both mechanical and biological benefits. Studies on the bioactivity of nano-sized HA and nano-titanium dioxide (n-TiO_2_) reinforced PEEK have been reported. Li *et al.* [98] fabricated HA/PEEK nanocomposites containing 15.1, 21.6, 29.2 and 38.2 vol % nano-sized HA (nHA) by powder processing and sintering. The tensile strength and fracture strain of PEEK nanocomposites filled with 21.6 and 29.2 vol % nHA match closely with those of human cortical bone. The results of SBF immersion, cell adhesion and proliferation *in vitro* suggested that 29.2 vol % nHA/PEEK nanocomposite possessed better bioactivity and biocompatibility than the other specimens. Wu *et al.* [99] fabricated n-TiO_2_ reinforced PEEK composites (n-TiO_2_/PEEK) and studied the bioactivity of these nanocomposites *in vitro* and *in vivo*. The effect of surface morphology or roughness was also considered. *In vitro* tests showed that n-TiO_2_ promoted cell attachment and improved osteoblast spreading. *In vivo* tests showed that n-TiO_2_ improved bone regeneration around the implants compared with pure PEEK, as assessed by micro-CT and histological analysis. Thus, n-TiO_2_ was considered to significantly improve the bioactivity of PEEK, especially for composites with rough surfaces.

## 4. Conclusions and Outlooks

PEEK is biocompatible, chemically and physically stable, radiolucent and exhibits a similar elastic modulus to normal human bone, making it an attractive orthopedic implant material. However, PEEK is biologically inert, preventing good bonding with surrounding bone tissue when it is implanted *in vivo*. Surface modification and composite preparation are two main strategies to improve the bioactivity of PEEK. For surface modification, including surface chemical treatment, physical treatment, and surface coating, the stability of the modified surface will be the key issue requiring further investigation. For the preparation of bioactive PEEK composites, the main challenge is to keep the excellent mechanical properties of PEEK when impregnating bioactive materials. The development of PEEK composites containing nano-sized bioactive materials may provide an effective way to obtain both mechanical and biological benefits.

## Figures and Tables

**Table 1.** The reported PEEK composites that are concerned with the bioactivity of the composites.

PEEK composites	Fillers (name, size, form)	Processing techniques	Research results related the bioactivity of the composites	Reference
HA/PEEK	Conventional HA particles	Melt compounding, granulation and injection molding	N/R	[63,64,66,67]
HA/PEEK	Conventional HA whiskers	Powder processing and compression molding	N/R	[65]
HA/PEEK	Conventional HA particles	Selective laser sintering	Cell tests: with improved osteoblast growth compared to TMX and PVC; higher HA contents with enhanced cell proliferation and osteogenic differentiation.	[68]
HA/PEEK	Conventional HA powders	*In situ* synthetic process	*In vivo*: the new bone tissues surrounding the composite grow faster with a higher HA content.	[69,70]
HA/PEEK	Conventional HA powders	Mixing, compaction and pressureless sintering	SBF immersion test: the 40 vol %-HA composite was covered by apatite-layer after 3 days; the growth rate increased with HA volume fraction.	[72]
Porous HA/PEEK	Conventional HA particles	Leaching of particulate technique	*In vivo*: formation of fibro-vascular tissue within the pores at 6 weeks and mature bone at 16 weeks.	[66]
Porous HA/PEEK	Conventional HA powers	Selective laser sintering	SBF immersion test and cell tests: with precipitation of apatite-layers; with positive cell adhesion and growth compared to control (no specimens).	[74,75]
Sr-HA/ PEEK	Conventional Sr-HA powers	Mixing, compression and molding	SBF immersion test and cell tests: with improved apatite-formation ability and mineralization compared to HA/PEEK or pure PEEK.	[77]
CS/PEEK	Conventional CS powers	Mixing and compaction	SBF immersion test: except for pure PEEK, all of the CS-containing composites promoted apatite-formation.	[78]
Glass/PEEK	Conventional Chopped E-glass fibers	N/A	Cell tests: with improved cell proliferation, ALP activity and OC production compared to polystyrene.	[79]
β-TCP/PEEK	Conventional β-TCP powers	Injection and molding	Cell tests: with inhibited cell proliferation, but with no concentration-dependent decrease.	[83]
Carbon black/ β-TCP/PEEK	Nano-sized carbon black powers, Conventional β-TCP powers	Laser sintering	Cell tests: with no improvement of cell proliferation compared to pure PEEK and carbon black/PEEK.	[81,82]
Carbon black/ bioglass/PEEK	Nano-sized carbon black powers, Conventional bioglass powers	Laser sintering	Cell tests: with improvement of cell proliferation compared to PEEK, carbon black/PEEK, and carbon black/β-TCP/PEEK.	[82]
HA/PEEK	Nano-sized HA particles	Compounding and injection molding	N/R	[84,85]
HA/PEEK	Nano-sized HA particles	*In situ* synthetic process	N/R	[88]
HA/PEEK	Nano-sized HA rods	Powder processing and sintering	Cell tests: with improved apatite-formation ability, cell adhesion and proliferation compared to pure PEEK.	[98]
TiO_2_/PEEK	Nano-sized TiO_2_ particles	Mixing compression and molding	Cell tests: with improved cell attachment and spreading compared with pure PEEK; *In vivo*: with improved bone regeneration around the implants compared to pure PEEK.	[99]

N/A, not applicable; N/R, not report.
